# Assessing the Country-Level Excess All-Cause Mortality and the Impacts of Air Pollution and Human Activity during the COVID-19 Epidemic

**DOI:** 10.3390/ijerph18136883

**Published:** 2021-06-26

**Authors:** Yuan Meng, Man Sing Wong, Hanfa Xing, Mei-Po Kwan, Rui Zhu

**Affiliations:** 1 Department of Land Surveying and Geo-Informatics, The Hong Kong Polytechnic University, Hong Kong; myuan.meng@connect.polyu.hk (Y.M.); felix.zhu@polyu.edu.hk (R.Z.); 2 Research Institute for Sustainable Urban Development, The Hong Kong Polytechnic University, Hong Kong; 3 School of Geography, South China Normal University, Guangzhou 510000, China; xinghanfa@sdnu.edu.cn; 4 College of Geography and Environment, Shandong Normal University, Jinan 250000, China; 5 Department of Geography and Resource Management, The Chinese University of Hong Kong, Hong Kong; mpk654@gmail.com; 6 Institute of Space and Earth Information Science, The Chinese University of Hong Kong, Hong Kong; 7 Department of Human Geography and Spatial Planning, Utrecht University, 3584 CB Utrecht, The Netherlands

**Keywords:** excess mortality, air pollution, human activities, COVID-19 mortality, NO_2_, PM_2.5_

## Abstract

The impact of Coronavirus Disease 2019 (COVID-19) on cause-specific mortality has been investigated on a global scale. However, less is known about the excess all-cause mortality and air pollution-human activity responses. This study estimated the weekly excess all-cause mortality during COVID-19 and evaluated the impacts of air pollution and human activities on mortality variations during the 10th to 52nd weeks of 2020 among sixteen countries. A SARIMA model was adopted to estimate the mortality benchmark based on short-term mortality during 2015–2019 and calculate excess mortality. A quasi-likelihood Poisson-based GAM model was further applied for air pollution/human activity response evaluation, namely ground-level NO_2_ and PM_2.5_ and the visit frequencies of parks and workplaces. The findings showed that, compared with COVID-19 mortality (i.e., cause-specific mortality), excess all-cause mortality changed from −26.52% to 373.60% during the 10th to 52nd weeks across the sixteen countries examined, revealing higher excess all-cause mortality than COVID-19 mortality in most countries. For the impact of air pollution and human activities, the average country-level relative risk showed that one unit increase in weekly NO_2_, PM_2.5_, park visits and workplace visits was associated with approximately 1.54% increase and 0.19%, 0.23%, and 0.23% decrease in excess all-cause mortality, respectively. Moreover, compared with the impact on COVID-19 mortality, the relative risks of weekly NO_2_ and PM_2.5_ were lower, and the relative risks of weekly park and workplace visits were higher for excess all-cause mortality. These results suggest that the estimation based on excess all-cause mortality reduced the potential impact of air pollution and enhanced the influence of human activities compared with the estimation based on COVID-19 mortality.

## 1. Introduction

Coronavirus Disease 2019 (COVID-19), caused by severe acute respiratory syndrome coronavirus 2 (SARS-CoV-2), was first identified in December 2019, and has caused 2,695,004 deaths worldwide as of 18 March 2021. Countries around the world proposed many policies to mitigate the spread of the disease, including lockdowns and the restriction of human mobility [1,2]. However, the lockdown policies themselves may have triggered other health issues. Research revealed that the restriction of social contact can cause severe mental health issues [3,4,5] and physical problems such as obesity [6]. In fact, there were death cases around the world from people who were at high risk of other diseases (e.g., cancer) who suffered from COVID-19. The data may have led to biases in COVID-19 death rate evaluations—which have been defined herein as excess all-cause mortality [7]. The reason for the present work’s focus on the excess all-cause mortality was to assess the variation between the number of deaths that occurred and the number of expected deaths in the absence of COVID-19 [8,9]. When compared with the death counts of COVID-19 (i.e., cause-specific mortality), excess all-cause mortality should depict the influence and impact of the COVID-19 epidemic with lower biases [10].

Excess all-cause mortality during COVID-19 was elucidated by researchers in many countries. For instance, Modi, et al. [11] indicated that excess mortality was approximately two times higher than COVID-19 deaths up until April 2020. Similar findings were found in the United States during the first three months of COVID-19, suggesting overstated excess mortality, compared with the official COVID-19 mortality [12]. In Germany, the excess mortality rate varied temporally in different weeks [13]. Moreover, a significant association was found between excess mortality and older-age groups [14,15]. The differences in excess mortality were also correlated by gender, with more men than women dying from COVID-19 [16,17]. Considering its spatial and temporal variation in different countries, it was deemed necessary to collate the excess all-cause mortality with fine temporal scale—and on a global scale.

Current research estimating the excess all-cause mortality associated with COVID-19 can be divided into two approaches. The first approach consists of calculating the average death rate over the past several years [8,18]. The second approach involves estimating excess mortality based on statistical models. For instance, Gibertoni et al. [15] estimated the number of observed deaths using time as an independent variable based on a linear regression model. General additional models using weeks of the year as indicator functions have been adopted to establish a semiparametric model for excess mortality estimation [12]. Moreover, spatiotemporal trends have been considered as a method to form a simulation-based approach for mortality count prediction [19]. Researchers have also used various transmission suppression levels and different relative risks to estimate excess mortality based on different COVID-19 incidence scenarios [20]. However, differences in estimation accuracy among these approaches remain unclear. Furthermore, to what extent the excess all-cause mortality was influenced by the COVID-19 outbreak on a global scale is also unknown.

Research has suggested that rising COVID-19 cases vary not only by demographic characteristics but also by environmental and human activity-related conditions [21,22,23,24]. Air pollutants, including nitrogen dioxide (NO_2_) and fine particulate matter with a diameter less than 2.5 μm (PM_2.5_), have been shown to be ambient anthropogenic emissions associated with acute respiratory infections and asthma cases [25,26]. Researchers evaluated the potential impact of decreasing NO_2_ and PM_2.5_ levels—which were associated with declining COVID-19 mortality trends [27,28]. The results revealed that chronic exposure to air pollutants delays the recovery period after COVID-19 infection and leads to more severe conditions [29,30]. Meanwhile, regular human activity patterns, including work and leisure, were significantly changed due to restrictions on social contact. Those restrictions, in turn, influenced the transmission rates of COVID-19 [31,32]. Research also suggested that green spaces facilitate social distancing, and could potentially mitigate the spread of COVID-19 [33]. In addition, growing trends related to working from home could be assessed in view of the incidence of COVID-19 [34]. Despite the fact that significant research has been done on the environmental and socioeconomic responses to COVID-19 incidence and mortality, their potential impact on the excess all-cause mortality required deeper investigation.

Faced with these challenges, this study estimated the excess all-cause mortality during COVID-19 and further investigated the potential impact of air pollution and human activity in 16 countries. Specifically, weekly excess deaths were estimated and compared by proposing three-year average mortality and Seasonal Autoregressive Integrated Moving Average (SARIMA) model-based mortality estimations. These were further evaluated by investigating the potential influence of exposure to NO_2_ and PM_2.5_ and the frequency with which people visited parks and workplaces. This study could help policymakers and stakeholders to better understand the COVID-19 pandemic under high-risk conditions.

## 2. Materials and Methods

### 2.1. Study Area and Datasets

This study investigated the excess all-cause mortality and the influence of air pollution and human activity based on the country level, and selected sixteen countries as its study area, including: Belgium, Chile, Croatia, the Czech Republic, France, Germany, Hungary, Lithuania, Luxembourg, Norway, Poland, Spain, Switzerland, and the United States.

We collected weekly country-wide all-cause mortality from the Short-Term Mortality Fluctuations data (STMF) in the Human Mortality Database (HMD) from the years 2015 to 2020. While weekly mortality rates from the 10th through the 52nd week during COVID-19 in 2020 were considered observed data, the mortality data from 2015 to 2019 were utilized to estimate the benchmark of weekly mortality without COVID-19 in 2020. On this basis, the excess all-cause mortality was considered to be the difference between the observed and benchmark mortality rates in 2020. Accordingly, the following quantification of the COVID-19 mortality, air pollution and human activities were all within the same time period: from the 10th through 52nd week, based on one-week intervals.

The COVID-19 mortality data were collected from the Johns Hopkins University Center for Systems Science and Engineering (JHU CSSE). Daily mortality data were summarized into weekly data to meet the time consistency of the weekly all-cause mortality.

The air pollution—including NO_2_ and PM_2.5_—was quantified using ground station data obtained from the OpenAQ Platform. The independent ground station data were aggregated into country-level data based on the population-weighted means method, in which global population data in 2019 from LandScan were utilized within the 10km buffers of air stations. The obtained air pollutants were first aggregated into daily data by filling missing values based on KalmanSmoother [35] and were subsequently summed into weekly data.

We focused on two aspects of human activity changes: business and leisure. In this study, we adapted data on the frequencies of daily visits to parks and workplaces from the Google community mobility report to depict human activity patterns. The quantification of the visit frequency values was presented as positive and negative percentages compared with the baseline day, calculated with the median value from a 5-week period during January and February in 2020. Daily human activities were aggregated into weekly data by summing the frequencies of daily visits to parks and workplaces.

In addition, we adapted meteorological data from the Global Surface Summary of the Day (GSOD) from the Integrated Surface Hourly (ISH) dataset, including daily mean temperature and total precipitation, as controlling data. Weekly data were aggregated based on the average daily temperature and the total daily precipitation in each week.

### 2.2. Excess Mortality Estimation

Excess mortality was defined as the difference between observed and benchmark death counts. While the observed mortality was the actual death count during a time period, the benchmark mortality was defined as the estimated death count according to historical trends, excluding the influence of unexpected disease (i.e., COVID-19).

To estimate the excess all-cause mortality during COVID-19 in each country, this study adopted two approaches to quantifying the benchmark mortality trends from the 10th to the 52nd week of 2020. The first approach was to estimate the benchmark death count based on three-year average mortality rates. Specifically, the average weekly death count within the predefined time period was calculated, and this was considered the benchmark based on the counterfactual condition of COVID-19.

The second approach was to predict the benchmark death count using a Seasonal Autoregressive Integrated Moving Average (SARIMA) model based on historical mortality trends from 2015 to 2019. The SARIMA model is presented as follows:

(1) $$SARIMA\left(p,d,q\right){\left(P,D,Q\right)}_{t}\;$$

where p,d,q, P,D,Q indicate, respectively: autoregressive order, difference order, and moving average order, seasonal autoregressive order, difference order, and moving average order. The proposed SARIMA models—with varied parameters—were estimated using the Kwiatkowski–Phillips–Schmidt–Shin (KPSS) tests, Bayesian information criterion (BIC) and the Ljung–Box test—the last of which was used to test the randomness of the temporal data. BIC was applied to estimate parameters (including p,q, P,Q) for better model performance. KPSS was performed to estimate the parameters *d* and *D*. It should be noted that the estimated values based on the SARIMA model were converted into death counts, with negative values converted into 0.

To evaluate the performance of the above two approaches among 16 selected countries, two indicators, including average error and the percentage of average errors lower than 0.1, were proposed. The average error in the *i*th country is calculated as follows:

(2) $$Predicted\;error_{i,t}=\frac{Actual\;death_{i,t}-Predicted\;death_{i,t}}{Actual\;death_{i,t}}$$

(3) $$Average\;error_{i}=\left|\overset{n}{\underset{t=1}{\sum }}Predicted\;error_{i,t}/n\right|\;$$

in which $Predicted\;error_{i,t}$ refers to the estimated error of death count in the *i*th country during the *t*th week. $Actual\;death_{i,t}$ and $Predicted\;death_{i,t}$ represent the actual and estimated death count in the *i*th country during the *t*th week. n is the total number of weeks. The percentage of average errors lower than 0.1 is calculated as:

(4) $$Percentage_{i}=n^{\prime }(\left|Predicted\;error_{i,t}\right|<0.1)/n.$$

where $n^{\prime }$ indicates the number of weeks with predicted errors lower than 0.1. Higher percentages are associated with higher accuracy. 

Considering the COVID-19 pandemic in 2020, the performances of both the three-year average mortality model and the SARIMA model were evaluated based on weekly mortality from 2015–2019, with data in 2015–2018 used for modelling and data in 2019 used for model assessment. Consequently, the predicted death count for 2020 was considered the benchmark and used to estimate differences with actual mortality in the same year.

### 2.3. Association Analysis

The impacts of air pollution and human activity on excess all-cause mortality in each country were estimated based on the quasi-likelihood Poisson-based GAM model. In particular, air pollution and human activities were quantified by weekly ground-based NO_2_ and PM_2.5_ levels and the frequency of visits to parks and workplaces. These were additionally controlled by weekly precipitation and temperature. Lag effects of 0, 1 and 2 weeks were used in the regression model to reduce the biases caused by time lagging. On this basis, interactions between weekly mortality and air pollution/human activity impacts were proposed to distinguish the differences between excess all-cause mortality and COVID-19 mortality in each country, modeled as follows:

(5) $$\log E\left(Y_{i}\right)=\alpha +\beta_{1}no2_{i,t}+\beta_{2}pm2.5_{i,t}+\beta_{3}park_{i,t}+\beta_{4}work_{i,t}+s\left(prec_{i,t}\right)+s\left(temp_{i,t}\right)$$

where $E\left(Y_{i}\right)$ refers to the expected weekly death counts in the *i*th country. Variables $no2_{i,t}$, $pm2.5_{i,t}$, $park_{i,t}$ and $work_{i,t}$ represent weekly ground-based NO_2_ and PM_2.5_ levels and the frequencies of visits to parks and workplaces in the *i*th country during the *t*th week, respectively. α indicates the intercept and $\beta_{1}$, $\beta_{2}$, $\beta_{3}$ and $\beta_{4}$ represent the coefficients of the corresponding variables. *s*() is the smoother, based on the penalized smoothing spline, with the number of knots being determined by generalized cross-validation (GCV).

## 3. Results

### 3.1. Excess All-Cause Mortality during COVID-19

**Figure 1:**
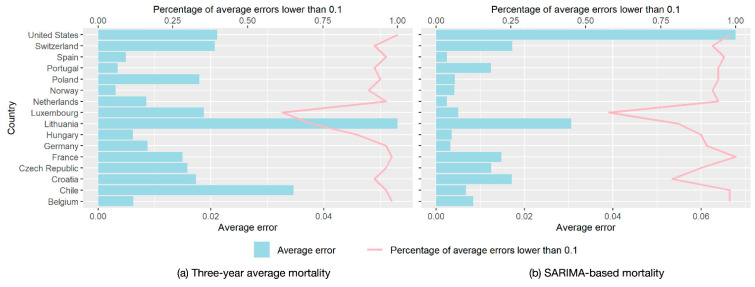
The performance of the three-year average mortality and SARIMA-based mortality estimations. (**a**) Three-year average mortality; (**b**) SARIMA-based mortality.

The performances of benchmark mortality based on the three-year average estimation and the SARIMA-based estimation are displayed in Figure 1. Average errors in most countries (based on the three-year average estimation) were below 0.02, while the average errors in SARIMA-based estimations were around 0.01. The percentages of average errors lower than 0.1 were approximately 0.9 for the three-year average estimation, while the percentages for SARIMA-based estimations were higher in most countries. Thus, we concluded that SARIMA-based estimation performed better than three-year average estimation in benchmark mortality estimation. The excess all-cause mortality during COVID-19 was further calculated based on the difference between the observed all-cause mortality and the benchmark all-cause mortality estimations based on the SARIMA model.

**Figure 2:**
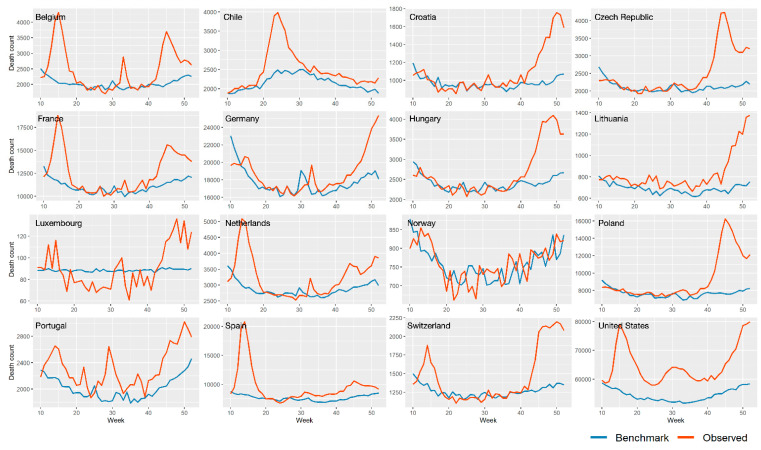
The all-cause observed and benchmark mortality during the 10th–52nd weeks in 2020 in 16 countries (The benchmark mortality is estimated by SARIMA model).

Figure 2 shows the observed and benchmark all-cause mortality rates over the 10th–52nd weeks of 2020 in 16 studied countries. Significant peaks of increasing death count in observed mortality rates were noted in comparison with the benchmark mortality in many countries. For instance, drastically increased mortality rates were seen in the differences between observed and benchmark data during the 20th to 30th weeks in Chile. Meanwhile, several peaks were observed during the 10th to 20th weeks, 30th to 35th weeks, and 40th to 52nd weeks in Belgium. On the other hand, continuously-increasing observed mortality rates (compared with the benchmark) were observed through the 10th–52nd weeks in the United States, indicating the large number of deaths caused by the COVID-19 outbreaks there.

**Figure 3:**
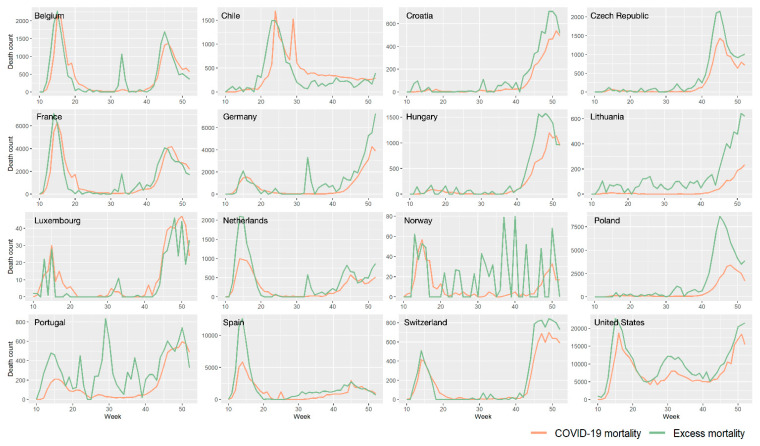
The excess and COVID-19 mortalities during the 10th–52nd weeks in 2020 in 16 countries. Excess mortality is calculated based on the benchmark estimated by the SARIMA model.

Figure 3 shows temporal weekly mortality trends during the 10th–52nd weeks in 16 countries, in order to further distinguish the excess all-cause mortality. Diverse temporal mortality patterns were observed in different countries. In 12 countries—Chile, Croatia, the Czech Republic, France, Hungary, Lithuania, Luxembourg, Netherlands, Poland, Spain, Switzerland and the United States—the variations observed in excess all-cause mortality were consistent with those of COVID-19 mortality. Essentially, the death count of excess all-cause mortality was significantly larger than COVID-19 mortality. This indicated that the outbreaks of COVID-19 had a drastic influence on increasing all-cause mortality. On the other hand, additional peaks of increasing death count for excess all-cause mortality were noted in four countries: Belgium, Germany, Norway and Portugal. The inconsistency between all-cause and COVID-19 mortality can be explained by the potential impact of COVID-19. For instance, studies have reported an increased risk of death in hospitalized COVID-19 patients with cancers, fever, or acute respiratory distress syndrome [36,37,38,39].

Table 1 summarizes the COVID-19 and excess all-cause death counts and the percentages of changes for all 16 countries. Chile and Luxembourg showed 12.94% and 26.52% decreases from COVID-19 to excess mortalities, respectively. In all remaining countries, increasing death counts were observed, ranging from 1.81% to 373.60%. This indicated the significant discrepancy between COVID-19 and excess mortalities, suggesting the need to investigate excess all-cause mortality during COVID-19 and the potential impacts of environmental and socioeconomic conditions in facilitating control of the virus.

### 3.2. Air Pollution/Human Activity Impacts Analysis

**Figure 4:**
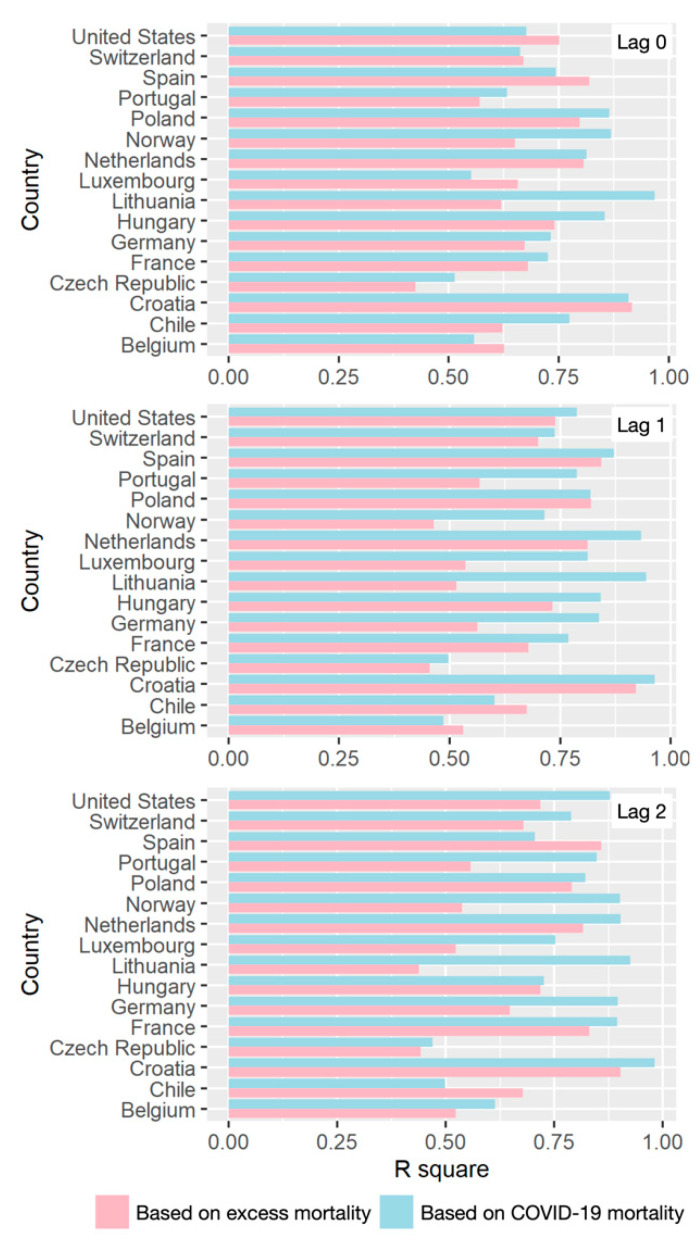
Comparison of R square values of GAM, based on excess mortality and COVID-19 mortality with lag effects of 0,1, and 2 weeks.

Table 2 summarizes the minimum, maximum, mean, and standard deviation values of air pollution data, human activity data, meteorological data, and excess all-cause mortality. On this basis, the impacts of air pollution and human activity were estimated on weekly excess all-cause mortality and COVID-19 mortality using the quasi-likelihood Poisson-based GAM model. The performances of GAM with lag effects of 0, 1, and 2 weeks were evaluated based on R square values. As shown in Figure 4, the performances of models based on COVID-19 mortality were relatively higher than those based on excess all-cause mortality. Moreover, no significances were observed among the models with different lag effects, with most values within 0.5–0.9. Model performances also varied among different countries; Croatia obtained relatively higher R square values than others. Considering that the R square values of most countries were higher than 0.5, the performances of models based on both excess and COVID-19 mortality were ensured.

**Figure 5:**
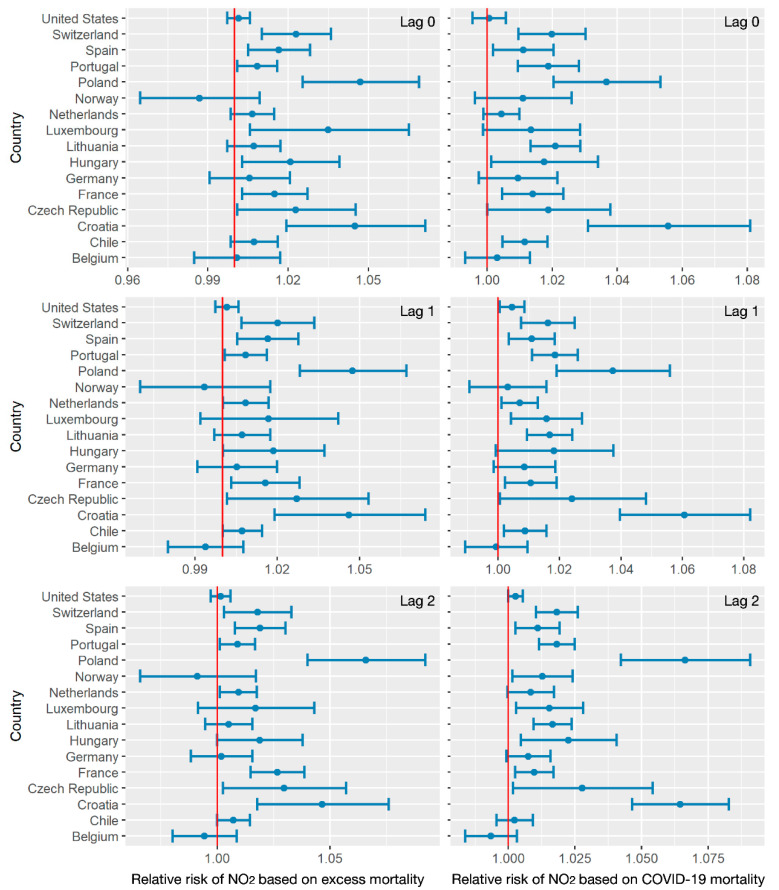
The relative risks of NO_2_ related to excess mortality and COVID-19 mortality, with lag effects of 0,1, and 2 weeks.

To distinguish the impacts of air pollution and human activities on both excess all-cause and COVID-19 mortalities, relative risks were calculated for each factor with a 95% confidence interval. Figure 5 shows the relative risks of weekly NO_2_ on the death count variation with lag effects of 0,1, and 2 weeks. For the estimation based on the excess all-cause mortality, relative risks varied from 0.99 to 1.05 in most countries. Lag effects showed less impact on the relative risk estimation of NO_2_ in most countries. However, the lag effects (1 or 2 weeks) in Belgium indicated a decreasing trend of NO_2_ on the increasing excess mortality, while less impact was observed with no lag effect. For the estimation based on COVID-19 mortality, the relative risks of NO_2_ were approximately within 1 to 1.08, suggesting that increasing NO_2_ levels corresponded to increasing COVID-19 mortality in most countries. Similar to the excess mortality-based estimation, the relative risks in Belgium also revealed negative patterns with lag effects. Compared with the COVID-19 mortality estimation, the relative risks of NO_2_ estimated by excess mortality were lower, suggesting less impact of NO_2_ when estimating potential environmental factors. On the other hand, despite the lockdown policies—which significantly reduced air pollutant emissions—the death counts of both excess and COVID-19 mortality were still positively related to ground-level NO_2_ emissions.

**Figure 6:**
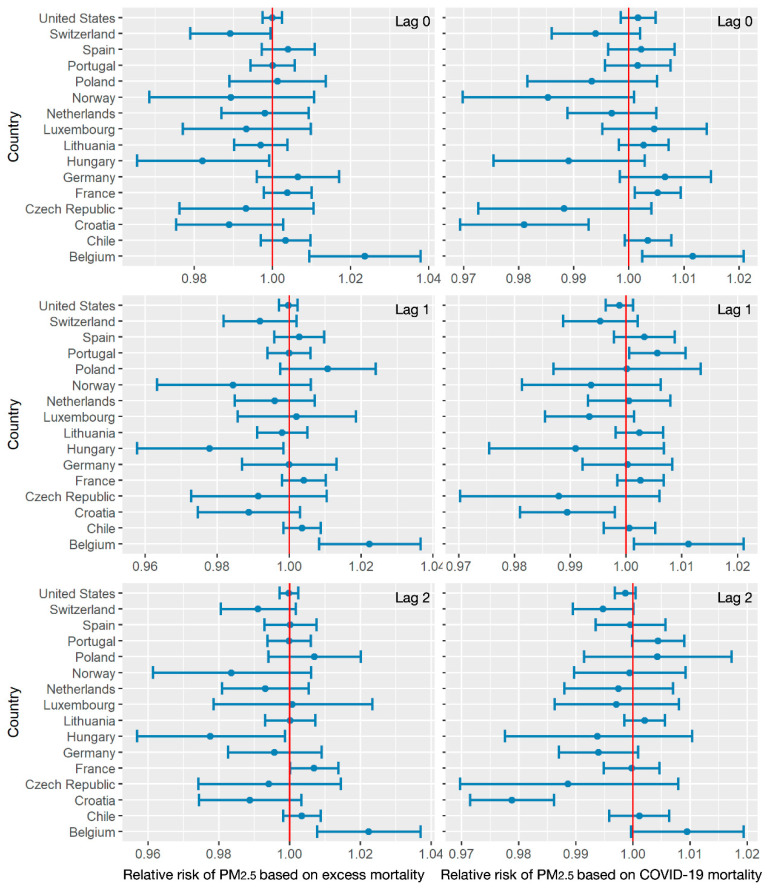
The relative risks of PM_2.5_ related to excess mortality and COVID-19 mortality with lag effects of 0,1 and 2 weeks.

Figure 6 shows the relative risks of PM_2.5_ based on excess all-cause and COVID-19 mortalities. While the ranges of relative risks were approximately within 0.96 to 1.04 (based on excess mortality), the relative risks based on COVID-19 narrowed down to 0.97–1.02. This indicated that the impact of PM_2.5_ on COVID-19 mortality has been overestimated compared with excess mortality. For instance, in Lithuania, Luxembourg, Portugal and the United States, no significant relative risks were observed with a 2-week lag effect based on excess mortality, whereas positive and negative relative risks from 0.99 to 1.01 were estimated in those countries based on COVID-19 mortality with a 2-week lag effect. Overall, lower levels of PM_2.5_ corresponded to increasing death counts in most countries. Additionally, 1- or 2-week lag effects increased these potential risks.

**Figure 7:**
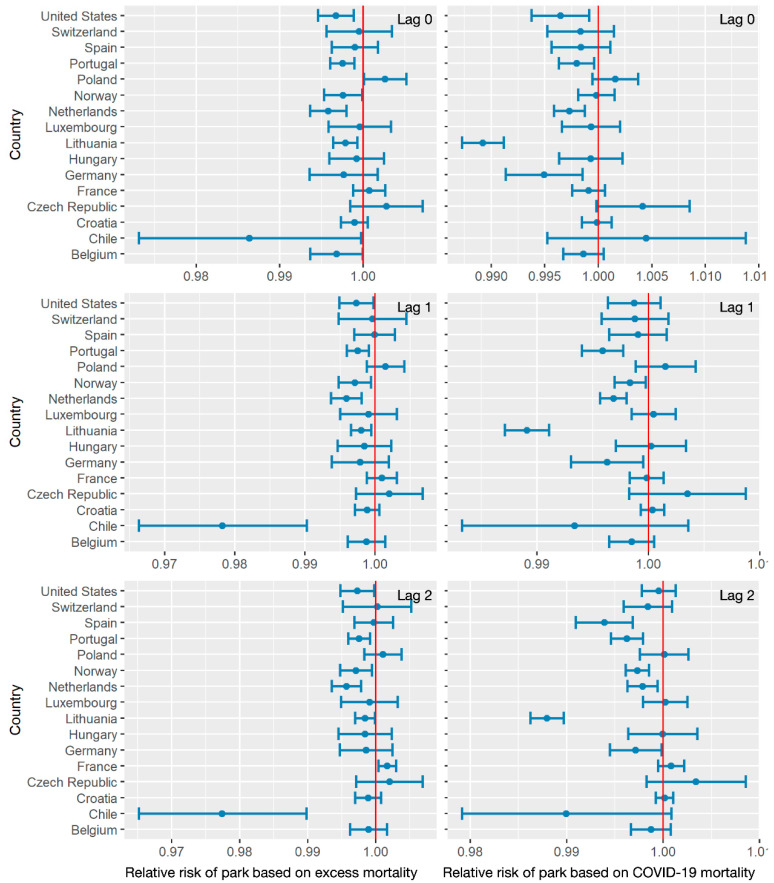
The relative risks of park visit frequency, related to excess mortality and COVID-19 mortality with lag effects of 0,1, and 2 weeks.

Figure 7 displays the influence of the frequencies of park visits on mortality variation. In particular, the relative risks in Chile showed significant differences from those in other countries. While relative risks—ranging from 0.995 to 1.01, based on COVID-19 mortality with no lag effect—revealed an overall positive trend between park visits and death counts, lower frequencies of park visits were related to increasing death counts when estimated based on excess mortality with lag effects of 0,1, and 2 weeks and when estimated based on COVID-19 mortality with lag effects of 1 and 2 weeks. A negative association was also revealed in the relationship between park visits and COVID-19 mortality in Lithuania. For other countries, higher relative risks of park visits were related to excess all-cause mortality with negative association—compared with those based on COVID-19 mortality with lag effects of 0,1, and 2 weeks. Specifically, a 1-unit decrease in park visits corresponded to approximately 1% increase in death count for excess mortality. 

**Figure 8:**
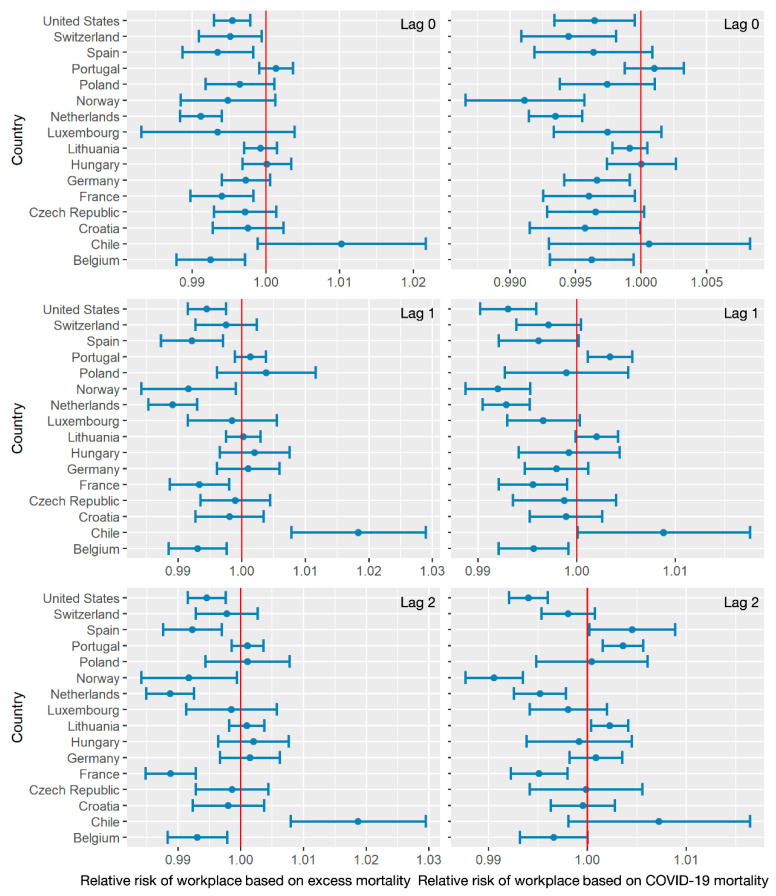
The relative risks of workplace visitation frequency, related to excess mortality and COVID-19 mortality, with lag effects of 0,1, and 2 weeks.

Figure 8 reveals the relative risks of workplace visitation to the excess all-cause and COVID-19 mortalities. Similar to park visits, the relative risks based on excess all-cause mortality were higher than those based on COVID-19 mortality. In terms of the excess all-cause mortality estimation, the relative risks in Chile showed a positive association between workplace visits and death count, ranging from 1 to 1.03. On the other hand, relative risks in other countries ranged from approximately 0.99 to 1 with lag effects of 0,1, and 2 weeks. This indicated a negative association between workplace visits and death counts in most countries. For the estimation based on COVID-19 mortality, a lesser influence was noted for workplace visits (approximately 0.995 to 1.005). The relative risks based on COVID-19 mortality also varied among studied countries, changing between negative and positive trends with lag effects in several countries, including Poland, Spain, and Luxembourg. On the other hand, consistent positive trends of relative risks were shown among different lag effects in some countries, including Chile and Portugal.

Table 3 displays the average country-level relative risks (without 95% confidence interval) of weekly NO_2_, PM_2.5_, park visits, and workplace visits on COVID-19 and excess mortality, with lag effects of 0, 1, and 2 weeks. Generally, positive relationships were revealed between weekly NO_2_ and COVID-19/excess mortality, while negative associations were shown between weekly PM_2.5_, park visits, workplace visits and COVID-19/excess mortality with lag effects of 0, 1, and 2 weeks. Mean relative risks of four variables (calculated based on the average relative risk with lag effects of 0, 1, and 2 weeks) showed values of 1.0171, 0.998, 0.9981, and 0.9979, associated with COVID-19 mortality, and values of 1.0154, 0.9981, 0.9977, and 0.9977, associated with excess all-cause mortality. Regardless of the positive or negative directions, the data suggested that weekly NO_2_ and PM_2.5_ levels were associated with lower relative risks for excess mortality than COVID-19 mortality—and that weekly park and workplace visits were associated with higher relative risks for excess mortality than COVID-19 mortality. 

## 4. Discussion

This study estimated the excess all-cause mortality during COVID-19 and found differences in the impacts of air pollution and human activity. Because of lockdowns and social distancing policies put in place to control COVID-19 transmission, anthropogenic emissions and regular human activities in many countries were significantly influenced. In particular, levels of air pollutants—including NO_2_ and PM_2.5_, which are considered major sources of anthropogenic emissions in public health issues—were altered. Meanwhile, researchers have discussed the potential impacts of working locations and parks due to movement restrictions [40,41]. This study adapted data on exposure to NO_2_ and PM_2.5_ and the frequencies of visits to parks and workplaces to depict variances in air pollution and human activity patterns. The findings revealed that the analysis based on excess all-cause mortality reduced the potential impact of NO_2_ and PM_2.5_ emissions and enhanced the influence of visits to parks and workplaces, as compared with the estimation based on COVID-19 mortality.

Although movement restrictions significantly decreased NO_2_ emissions, a positive association was still observed in NO_2_ related to excess mortality. Opposite trends were revealed for the association between PM_2.5_ and excess mortality in most countries. It should be noted that the findings of this study were not consistent with many other studies, which suggested an inverse association between exposure to NO_2_ and mortality and a positive association between PM_2.5_ and mortality [42,43]. The negative association between PM_2.5_ and excess mortality could be attributed to lockdown protocols and restricted social distancing policies, which significantly changed air pollutant emissions. In addition, the association discrepancies may be due to the heterogeneity of environmental and socioeconomic conditions among the studied countries. Controlling factors—such as meteorological and demographic patterns—could also change the accuracy of impact estimation. The use of different statistical methods in different studies is another factor that could cause estimation bias [44]. The inverse association between visits to parks and workplaces and excess all-cause mortality is in line with governmental response policies.

Limitations exist in this study. First, the inconsistency of COVID-19 mortality data and all-cause mortality data among countries should be considered. The statistics of COVID-19 cases and all-cause mortality cases varied widely among the studied countries, which could lead to biased estimations of excess all-cause mortality in specific countries. Incomplete COVID-19 cases in many countries could also cause this issue. Moreover, the number of deaths classified as COVID-19 deaths which may have been attributable to other high-risk conditions is still uncertain. While many countries, faced with this issue, have tended to attribute deaths to COVID-19, there are also countries that consider other underlying causes of death in addition to COVID-19 [10]. Second, due to the limited availability of weekly all-cause mortality data, this study focused on country-level analyses and ignored the heterogeneity of air pollution and human activities within countries. Thus, this study mainly investigated variations in air pollution, human activity, and excess mortality responses among countries, and compared the differences between excess all-cause mortality and COVID-19 mortality. Results suggested that it would be necessary to calculate excess all-cause mortality on a finer scale in order to support the estimation of potential air pollution and human activity impacts on regional, national and global scales.

## 5. Conclusions

This study estimated excess all-cause mortality during COVID-19, based on a SARIMA model, and further investigated the impacts of air pollution and human activity on mortality variations in sixteen countries using a quasi-likelihood Poisson-based GAM model. The results showed that COVID-19 dramatically increased death counts compared with benchmark mortality trends, leading to high rates of excess all-cause mortality. Moreover, the excess all-cause mortality changed from −26.52% to 373.60% when compared with COVID-19 mortality during the 10th–52nd weeks of 2020 in sixteen countries. For the impacts of air pollution and human activity, an increase of 1.54% and decreases of 0.19%, 0.23%, and 0.23% in excess all-cause mortality rates were revealed with a 1-unit increase of weekly NO_2_, PM_2.5_, park visits, and workplace visits, respectively. In addition, compared with the estimated relative risks of COVID-19 mortality, lower relative risks for weekly NO_2_ and PM_2.5_, and higher relative risks for weekly park and workplace visits were shown for excess all-cause mortality. This suggests that, compared with excess all-cause mortality, COVID-19 mortality overestimated the impact of air pollution and underestimated the influence of human activity. These findings provide an alternative perspective for policymakers and stakeholders to better assess the COVID-19 pandemic.

## Figures and Tables

**Table 1:** COVID-19 and excess all-cause mortality during the 10th–52nd weeks in 2020.

Country	COVID-19 Mortality	Excess All-Cause Mortality	Variation
Belgium	19,317	19,956	3.31%
Chile	16,443	14,315	−12.94%
Croatia	3671	5640	53.64%
Czech Republic	11,058	17,224	55.76%
France	62,634	63,767	1.81%
Germany	30,366	50,385	65.93%
Hungary	9047	13,905	53.70%
Lithuania	1254	5939	373.60%
Luxembourg	460	338	−26.52%
Netherland	11,095	17,538	58.07%
Norway	421	829	96.91%
Poland	27,118	71,237	162.69%
Portugal	6619	13,352	101.72%
Spain	51,744	82,355	59.16%
Switzerland	7316	9106	24.47%
United States	333,278	445,242	33.59%

**Table 2:** Summary of ground-level air pollution data, human activity data, meteorological data, and excess all-cause mortality.

	Min.	Max.	Mean	SD
Weekly NO_2_ (μg/m^3^)	0.000	227.039	117.327	35.680
Weekly PM_2.5_ (μg/m^3^)	0.000	204.573	66.256	32.085
Weekly park visits	−582.000	2859.000	250.076	483.875
Weekly workplace visits	−526.000	86.000	−191.499	102.288
Daily precipitation (inch)	0.000	2.616	0.517	0.492
Daily temperature (℉)	27.313	80.231	55.111	11.324
Weekly excess death count	0	1.29 × 10^8^	3.61 × 10^6^	1.25 × 10^7^

**Table 3:** Average country-level relative risks of weekly NO_2_, PM_2.5_, park visits, and workplace visits.

		Weekly NO_2_	Weekly PM_2.5_	Weekly Park Visits	Weekly Workplace Visits
Lag 0	COVID-19 mortality	1.0166	0.9979	0.9987	0.9968
	Excess mortality	1.0155	0.9983	0.998	0.9968
Lag 1	COVID-19 mortality	1.0162	0.9985	0.9982	0.9979
	Excess mortality	1.0145	0.9983	0.9975	0.9983
Lag 2	COVID-19 mortality	1.0185	0.9977	0.9976	0.999
	Excess mortality	1.0162	0.9977	0.9976	0.9979
Mean	COVID-19 mortality	1.0171	0.998	0.9981	0.9979
	Excess mortality	1.0154	0.9981	0.9977	0.9977
